# Factors related to women’s use of health insurance cover in Navakholo, Kakamega County, Kenya: sub-county level results based on community household register

**DOI:** 10.1186/s12889-023-15270-1

**Published:** 2023-03-28

**Authors:** Rachel Ambalu, Sadiq Rashid, Saul Atwa, Mariam Otira, Lucia Ndolo, David Ojakaa

**Affiliations:** 1 Amref Health Africa (H.Q), P.O. Box 27691-00506, Nairobi, Kenya; 2 BRIM Research, P. O. Box 76100, 00508 Code, Yaya Towers, Nairobi, Kenya

**Keywords:** Universal Health Coverage, National Health Insurance Fund, Women of Reproductive Age, Household register, Mobile phone, Kenya

## Abstract

**Background:**

In concert with international commitments, the Government of Kenya identified Universal Health Coverage (UHC), mainly through the National Health Insurance Fund (NHIF), as one of its four priority agenda to enable its populations access health care without financial duress. Nevertheless, only about 19.5% of the Kenyan population is enrolled in any insurance health cover. Since 2016, Amref Health Africa and PharmAccess Foundation have been implementing the Innovative Partnership for Universal and Sustainable Healthcare (iPUSH) programme in Navakholo sub-county of Kakamega County. The main objective of this study is to examine use of health insurance cover among Women of Reproductive Age (WRA) in Navakholo sub-county, Kakamega County.

**Methods:**

We analysed data captured during household registration conducted in February 2021 which embraced a question on use of health insurance cover including NHIF. The dataset consisted 148,957 household members within 32,262 households, 310 villages, and 32 community health units. The data had been collected using mobile phones by trained Community Health Volunteers (CHVs) and transmitted using the Amref electronic data management platform and reposited in a server. Data were analysed through frequency distributions and logistic regression (descriptive and causal methods) using STATA software.

**Results:**

Insurance coverage, all providers included, in Navakholo sub-county stood at 11% among women aged 15–49 years. This is much lower than the national aggregate reported from sample surveys, but higher than the 7% found in the same survey for the region where Navakholo is situated. Social determinant variables – age, perceived condition of the household, and wealth ranking – are highly significant in the relationship with use of health insurance cover while measures of reproductive health and health vulnerability are not.

**Conclusion:**

In Navakholo sub-county of Western Kenya, all—health-insurance coverage is lower than the national aggregate estimated from sample surveys. Age, perception of household condition, and wealth ranking are very significantly related to use of a health insurance cover. Frequent household registrations should be conducted to help monitor the trends and impact of health insurance campaigns. Training – upstream and downstream – on community household registration and data processing should be conducted to arrive at better quality data.

## Background

Target 3.8 of the United Nation’s sustainable development goals [1], focuses among others on the achievement of UHC, including financial risk protection for all by the year 2030. Beyond percentage coverage of the population with essential health services, a key concern in monitoring achievements of UHC indicators is equity. The Government of Kenya on its part, towards fulfilling this international commitment identified UHC as one of its four priority agenda, with the aspiration that Kenyan populations would have access to health care without sinking into financial catastrophe [2]. Nevertheless, only about 19.6% of the population in the country has health insurance cover [3].

Between 2016 and 2021, the non-governmental organizations (NGOs) Amref Health Africa and PharmAccess Foundation, together with the County Government of Kakamega located in Western Kenya, jointly implemented iPUSH programme in Navakholo sub-county. The iPUSH programme broadly sought to map out, recruit and register households into the National Health Insurance Fund (NHIF). An entry point and key target of the iPUSH programme in households was women of reproductive age (WRAs). Under the programme, selected WRAs and their households would be covered for reproductive, maternal, new-born and child health (RMNCH) services once they were provided with NHIF cover. Hence the focus in this study on WRAs.

To support the planning, monitoring, and evaluation of this activity, a household registration and socio-economic mapping exercise was conducted in February 2021 by trained CHVs using mobile phones, with the data being transmitted in near real-time to the organization’s server using its Mobile-Jamii Afya Link (MJALi) electronic data management platform. The data were collected using the household register (MOH 513) and the socio-economic mapping tool from the National Safety Net Programme (NSNP). The two forms are available as additional file 1 and additional file 2 respectively. The main objective of this study is to examine use ownership of health insurance cover among Women of Reproductive Age (WRA) in Navakholo sub-county, Kakamega County in Kenya. The specific objectives are: (1) to examine the demographic, socio-economic, and health status characteristics of households and individual women of reproductive age (WRAs) in Navakholo sub-county in relation to health insurance cover; (2) determine the extent of health insurance equity; (3) determine the relation between use of health insurance cover and individual-level characteristics.

With a total population of 153,977 people and 32,315 households registered in the 2019 census [4], Navakholo sub-county is one among 13 sub-counties in Kakamega County of Western Kenya. The sub-county forms part of the central and northern part of the county and which is ecologically in the Upper Medium (UM) zone practicing intensive small-scale agriculture [5]. Compared to 35% of the Kenyan national population living below the poverty line in 2015, the average for Kakamega county and indicative of Navakholo sub-county was 33.3% [6]. The main diseases in Kakamega County which includes Navakholo sub-county, are malaria (with a prevalence of 19% against a national average of 6%) [7, 8] and those of the respiratory system.

A number of themes emerge in the literature on health insurance in Kenya and are briefly described below. The centrality of NHIF of Kenya and its planned transformation into a universal health entity is a key topic that runs in the literature [9]. Universal health coverage has been adopted as Target 3.8 of the Sustainable Development Goals (SDGs), with a clear goal of ensuring that individuals and communities receive the health services they need without suffering financial hardship. For health insurance coverage, analysis of secondary data from the Kenya Demographic and Health Survey (KDHS) shows that it is low in Kenya, at 19.6% of the population as of 2014 [2, 9]. This is against the back-drop of about 50% of the population living below the poverty line. Although there was a decrease in inequality in health insurance coverage between 2009 and 2014, levels of inequality remain high. It is older people, those in formal employment, the married, those exposed to mass media, the males, those belonging to a small household, those with a chronic disease, and those in well-to-do households who have an increased likelihood of health insurance coverage. As such, attaining high and equitable coverage with either contributory or voluntary insurance scheme is an issue. The study [9] calls for a universal, tax-funded programme in which ensures that revenues are efficiently and equitably collected; everyone including the poor and vulnerable should be covered.

The critical importance of UHC is demonstrated by its adoption by the Kenya Government as one of the big four priority agenda by the President [2]. The aspiration is that by 2022, all persons in Kenya will be able to use the essential services they need for their health and wellbeing through a single unified benefit package, without the risk of financial catastrophe. Results from Peru [10] a developing middle-income country, identify the impact of the progressive universalist policy effected in 2007 and which gave Peruvian adults entitlement to basic health services in public health facilities without charge, a service for which they previously were required to pay user fees. The evaluation of this policy was achieved by comparing the change in health care utilization among the target population with that of poorer adults already covered under employment-based insurance. Positive effects are evident after receiving outpatient care and medication; these are largest among the elderly and poorest populations. The likelihood of getting health care when sick is increased by almost 20%; the chances of being unable to afford treatment is reduced by almost 25%. There is no effect on average out of pocket expenditure (OOPE) but medical expenditure is reduced by almost 25% among the top 25% of the population. The study concludes that giving poor Peruvians entitlement to free basic health care was partially successful in targeting the poor with access to health care and protection from medical expenditure risks.

A household survey conducted in eight Kenya counties [11] documents the relationships between health insurance and medicine expenditure. This is against the background of the Kenya national and various County Governments initiating health insurance schemes to protect households from financial hardship resulting from large OOPE. While the demographic results are similar to those from other studies cited above, the different finding from the study is that households with health insurance cover have a lower likelihood of OOPE on medicine, and still less on medicines out of their total health expenditure. Like others, this study suggests prioritization of low-income households as well as those with non-communicable diseases in order to hasten access to medication and financial protection.

That social insurance is a viable option for improving access of the population to health services, as well as improving health outcomes for deprived populations in particular HIV-positive women is a subject covered in the literature [12]. The results of the study show that health insurance enrolment is related with increased utilization of obstetric services among HIV-positive women in the country. In particular, HIV-positive women have increased access to health-facility birth delivery and skilled birth attendance as compared to those who are uninsured. Positive effects of NHIF on use of obstetric care services is higher for those who are sicker (CD4 count of less than 350 cells/µL).

Review of the UHC pilot programmes in Isiolo and Kisumu counties captures learning to inform scale-up. In Isiolo and Kisumu counties, as in the other two of the total four pilot counties, the County governments discontinued charging user fees in secondary public health hospitals. In return, the national Government provided commodities and additional funds from the national Government. The process of implementing the pilot UHC programme is documented in the literature [13, 14] and the particular role of CHVs and the community emerges. In Kisumu, sensitization about the UHC programme was conducted through electronic media, by CHVs, education sessions, the political class and outreaches. Planning for the programme was implemented through meetings, training for community registration, and developing budgets.

In Isiolo, a number of achievements can be attributed to the UHC pilot programme [13]. The intervention reached a majority of Isiolo population thus enabling access to health services free of charge. The UHC funds flowed to the health facilities and used to improve infrastructure and provide better services. Stock-out of medicines and supplies reduced during the pilot programme.

Nevertheless, in Isiolo, concerning challenges, funds were received with a delay resulting in partial implementation of the pilot. Stakeholders suggested that a simplified process to access and use funds would lead to better performance. Steering committees and technical working groups could not be constituted or work due to lack of funds. Some health facilities had problems developing their work plan due to lack of guidance and templates; work plans were not always followed owing to shifting priorities at facility and county levels. Only a few staff were hired in the facilities resulting in increased workload, a barrier that was similarly documented for Kisumu County pilot.

In addition, from the review for Kisumu County [14], misunderstanding, confusion and misconception about the idea of UHC is evident. It was seen as a means of seeking votes by politicians. In Isiolo, there were also issues concerning the correctness of cardholders’ information; not all health facilities had a verification system in place. The review suggests that in future, counties should consider using the funds more strategically in order to influence the behavior of provider and address health needs of the population, for example through the application of performance-based financing (PBF) to incentivize health facilities and workers. Other studies of the Kenyan four-county pilot UHC programme [15] conclude that for utopian and egalitarian projects such as UHC, civil servants maintain hope and optimism even in the backdrop of their past experience with failure of such projects. The objective of this study is to examine the factors related to use of health insurance cover among WRAs in Navakholo sub-county, Kakamega County.

## Methods

This study conducted secondary analysis of data on household and socio-economic collected in February 2021 using form MOH 513 and the socio-economic mapping questionnaire respectively. During this time, it is assumed that there were no significant migration flows for the Sub- County, given its rural setting, lower economic mobility, and partial lockdown. For review and analysis, the data were downloaded from the Amref server using excel and transformed into the STATA statistical programme for further review, and descriptive as well as multivariate causal analysis. Python programme was also used to process particular variables in the data such as condition of the household, before analysis. In addition to the already available household condition variable in the data, we also constructed a wealth ranking variable using the household asset information in the data [16]. The main observations from the initial review of the data were that first, demographic variables such as population size and age, as well as household factors which included household size, wealth categories, and condition of the household were better reported. Secondly, nevertheless, data quality issues were encountered with a number of important socio-economic variables particularly marital status, educational level, and occupation. Specifically, the variables mentioned were missing for many records. As such these were not included in the analysis. Although community health volunteers were trained prior to data collection using the community household register, this is can be described as a routine and frequent upstream exercise not subject to the higher rigor in a formal research data collection exercise. Similarly, downstream at the stage of data processing the data downloaded from the internet server it is only now, with the appreciation of the value of this dataset, that stringent data quality controls may be beginning to be applied. So, this study also demonstrates the value of the data and hence the need to pay more attention to its quality.

## Results

### General population and household characteristics

Based on the household registration exercise conducted by CHVs, the total population for Navakholo sub-county in February 2021 was 148,957 people residing 32,262 households, 310 villages and 32 community health units (CHUs). This population is at least 3.3% lower than that registered in the 2019 Kenya census of population and hosing for the sub-county of 153,977 people in 32,315 households [3]. The undercount of the sub-county’s total population (which would be higher than the 3.3% above based on a projection of the 2019 census to February 2021) and number of households based on the household register could be due to missing to enumerate some villages, which are suspected to be more than the 310 derived from the household registration data.

**Fig. 1:**
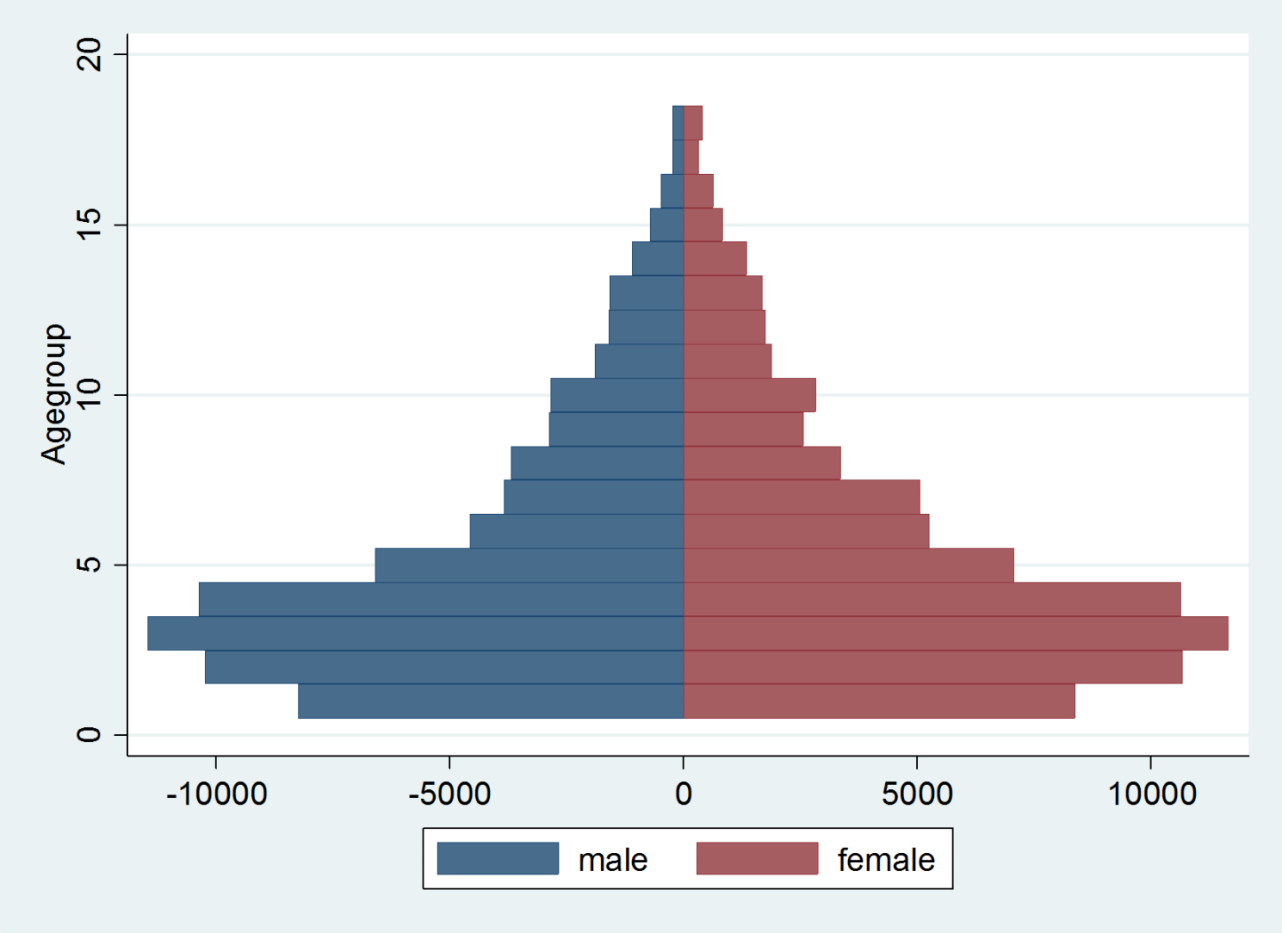
Population pyramid for Navakholo Sub-county based on community household registration, February 2021. (Legend for Age groups: 1 = 0–4; 2 = 5–9; 3 = 10–14; 4 = 15–19; 5 = 20–24; 6 = 25–29; 7 = 30–34; 8 = 35–39; 9 = 40–44; 10 = 45–49; 11 = 50–54; 12 = 55–59; 13 = 60–64; 14 = 65–69; 15 = 70–74; 16 = 75–79; 17 = 80–84; 18 = 85+)

An indication of the age composition of the population of Navakholo sub-county is shown in Fig. 1 below. It shows the usual youth bulge typical of developing countries with a smaller cohort of children aged 0–4 years usually attributed to transitions to reduced fertility. The youthful nature of the populations further indicated by the mean age of the total population which is 23.3 years. The population pyramid also shows the population of older people aged 65 years and above, with the oldest person being 115 years of age. The gender distribution also shows the slightly higher female sex ratio, with females making 51.2% of the population while men comprise the complement of 48.8%. The relationship to the household head in the whole population again shows the youthful nature of the population, as child and grandchild make up 52% and 10.1% respectively of the total population while the household heads comprise 21.6% of the whole population. The other relationships are spouse (14.5%), brother or sister (0.4%), and others (1.3%).

The distribution of household heads (who numbered 32,160, mean age 47.0 years and whose mean household size was 4.6 persons) by various characteristics is shown in Table 1 below.

**Table 1:** Household characteristics

Household size	Female		Percent		Male		Percent		Total		Percent	
1–2	2,964		34.2		3,554		15.1		6,518		20.3	
3–8	5,455		63.0		18,309		77.9		23,764		73.9	
9+	239		2.8		1,639		7.0		1,878		5.8	
Total	8,658		100.0		23,502		100.0		32,160		100.0	
**Wealth ranking**												
Lowest	1,836		21.2		4,645		19.8		6,481		20.2	
Second	2,234		25.8		4,200		17.9		6,434		20.0	
Third	1,984		22.9		4,460		19.0		6,444		20.0	
Fourth	1,206		13.9		5,251		22.3		6,457		20.1	
Fifth	1,398		16.2		4,946		21.1		6,344		19.7	
Total	8,658		100		23,502		100.0		32,160		100	
**Insurance cover**												
Yes	851		9.8		3,477		14.8		4,328		13.5	
No	7,807		90.2		20,025		85.2		27,832		86.5	
Total	8,658		100		23,502		100.0		32,160		100.0	
**Condition of Household**												
Poor	3,134		37.2		5,946		26.1		9,080		29.1	
Fair	4,515		53.6		13,959		61.3		18,474		59.2	
Good	696		8.3		2,598		11.4		3,294		10.6	
Very good	82		1.0		276		1.2		358		1.1	
Total	8,427		100.0		22,779		100.0		31,206		100.0	

The table shows that 13.5% of households in Navakholo own a health insurance cover (whether under the UHC scheme, directly with NHIF, or other (private insurance) cover. This percentage is higher among male-headed households (14.8%) and lower among female-headed households (9.8%). Overall, households with 3–8 persons are the most frequently occurring (7.9%) and the same pattern is to be observed when divisions are made by gender. Similarly, 59.2% of households think that their condition is fair while 29.1% of households think that their condition is poor. On the other hand, 10.6% think that their condition is good, while only 1.1% think that it is very good.

## Characteristics of individual women of reproductive age

Compared to all household heads, a smaller proportion (11%) of women (who are either spouses or heads of households) aged 15–49 years have a health insurance (Table 2). The proportion of women by age group rises from age 15–19 (1.9%) to a maximum in age group 30–34 (22.8) after which it reduces gradually to reach 13.3% in age group 45–49. Of these identified women aged 15–49, 82.3% are spouses while the rest (17.7%) are heads of households. A majority of the women (84.6%) are in households of 3–8 members, while only 6.9% are in households of more than 9 members, as 6.5% are in households with 1 to 2 members. The majority of the women (63.2%) described their household condition as fair.

**Table 2:** Characteristics for individual women of reproductive age

Health insurance cover	Number	Percentage	Cumulative percentage	
None	18,370	89.0	89.0	
Yes	2,267	11.0	100.0	
Total	20,637	100.0		
**Age group**				
15–19	400	1.9	1.9	
20–24	2,912	14.1	16.1	
25–29	4,200	20.4	36.4	
30–34	4,701	22.8	59.2	
35–39	3,199	15.5	74.7	
40–44	2,474	12.0	86.7	
45–49	2,751	13.3	100.0	
Total	20,637	100.0		
**Relationship**				
Household head	3,651	17.7	17.7	
Spouse	16,986	82.3	100.0	
Total	20,637	100.0		
**Size of household**				
1–2	1,744	8.5	8.5	
3–8	17,464	84.6	93.1	
9+	1,429	6.9	100.0	
Total	20,637	100.0		
**Wealth ranking**				
Lowest	4,674	22.7	22.7	
Second	3,458	16.8	39.5	
Third	3,684	17.9	57.4	
Fourth	4,528	22.0	79.3	
Fifth	4,259	20.7	100.0	
Total	20,603	100.0		
**Household condition**				
Poor	5,064	25.3	25.3	
Fair	12,663	63.2	88.4	
Good	2,139	10.7	99.1	
Very good	184	0.9	100.0	
Total	20,050	100.0		
**Pregnant**				
No	19,502	94.5		
Yes	1,135	5.5		
Total	20,637	100		
**Chronic disease**				
None	18,361	88.97		
Yes	2,276	11.03		
Total	20,637	100		

On the other hand, 25.3% indicated their household’s condition to be poor, 10.7% good, and 0.9% as very good. Regarding indicators for RMNCH, 5.5% of the women aged 15–49 years indicated that they were currently pregnant. To the extent that equity is a core indicator of UHC programmes, a factor in equity is whether disadvantaged, marginalized, and vulnerable populations have access to health insurance cover which would increase their chances of accessing health care services that they require. Consequently, whether a woman has a chronic disease or not was included as one of the background factors in this study. The results in Table 2 show that 11% of women 15–49 years old have a chronic disease which encompass diabetes, cancer, mental illness, hypertension, and chronic respiratory diseases as indicated in MOH 513 (additional file 1).

## Regression of use of health insurance cover on covariates

It had been hypothesized that various demographic and socio-economic background factors, as well as reproductive (whether pregnant) and health status (chronic disease) would influence owning a health insurance cover. The results of the regression are shown in Table 3 below.

**Table 3:** Logistic regression of health insurance cover use on selected covariates

Dep.=owns health insurance cover 0 = No 1 = Yes	Coef.	Odds ratio	Std. Err.	P > z	[95% Conf.	Interval]	Significance
**Age group** Ref.=15–19							
20–24	0.678	1.969	0.355	0.056	-0.019	1.374	
25–29	1.147	3.149	0.350	0.001	0.461	1.833	**
30–34	1.240	3.456	0.350	0.000	0.555	1.926	***
35–39	1.426	4.163	0.351	0.000	0.738	2.114	***
40–44	1.495	4.458	0.352	0.000	0.804	2.185	***
45–49	1.278	3.588	0.353	0.000	0.586	1.969	***
**Relationship** Ref.=Household head							
Spouse	0.084	1.087	0.069	0.223	-0.051	0.218	
**Size of household** Ref.**=**1–2							
3–8	0.067	1.070	0.105	0.521	-0.138	0.273	
9+	-0.235	0.790	0.141	0.096	-0.512	0.042	
**Wealth rank** Ref.=First							
Second	0.106	1.112	0.111	0.341	-0.112	0.324	
Third	0.526	1.692	0.101	0.000	0.327	0.725	***
Fourth	0.583	1.792	0.090	0.000	0.407	0.760	***
Fifth	1.457	4.291	0.084	0.000	1.291	1.622	***
**Household Condition** Ref.=Poor							
Fair	0.944	2.570	0.092	0.000	0.763	1.125	***
Good	1.913	6.774	0.104	0.000	1.710	2.116	***
Very good	2.500	12.186	0.179	0.000	2.149	2.851	***
**Pregnant** Ref.=No							
Yes	0.174	1.190	0.111	0.117	-0.044	0.392	
**Chronic disease** Ref.=No							
Yes	0.085	1.088	0.078	0.278	-0.068	0.238	
_cons	-5.168		0.370	0.000	-5.892	-4.443	

* significant at 5%; ** significant at 1% ; *** significant at 0.1%.

The results show that age is positively and very significantly related to use of health insurance cover. Thus, compared to age group 15–19, age 20–24 has a about 2 times odds of owning a health insurance cover. These increase steadily with age so that by age group 40–44, the odds are 4.4 after which it decreases to 3.6 for the age bracket 45–49. Wealth rank and perceived condition of the household are all significantly and positively related to use of health insurance cover. Thus, in particular, given poor household status, fair perception of the household gives rise to 2.570 odds of owning a health insurance cover. The odds increase to 6.774 for good household condition, jumping to 12.186 for the perception of very good household condition. The factor associated with RMNCH in this study, namely current pregnancy status, is not a significant predictor of owning a health insurance cover. Similarly, the indicator of vulnerability due to chronic disease is not significantly related to use of health insurance cover.

## Discussion

Informed in part by the idea of the social determinants of health [16] and the objectives of this study, this section discusses the findings around four themes: use of health insurance cover, social determinants, vulnerability, and reproductive status. Based on the household register, use of health insurance cover in 2021 among women aged 15–49 years in this rural sub-county in Western Kenya is much lower (at 11%) than the national sample aggregate of 18% (but higher than the average of 7% for the Western region) reported earlier for the year 2014 [8; 9]. Compared to the sample estimate above for Western region whose sample size is 1,571 women of reproductive age, the larger number of women in the household registration (20,367) provides an appealing confirmatory check. The results confirm findings of low health insurance cover in other countries in the Africa region such as Nigeria and South Africa where coverage was 2.7% and 12.8% respectively [17].

For variables grouped under social determinants of health, increasing age, income, self-perception on condition of the household are very statistically significant. These findings resonate well with results from elsewhere in Africa such as Ghana and Kenya [18, 19] which find that older women are more likely to take up health insurance. Similar to this study, the literature [2, 9] also confirms the strong influence income plays as a covariate in the uptake of health insurance. Although being a spouse as compared to being the household head is related to increased uptake of health insurance cover, with a marginal increased odds ratio of 1.087, it is not significant. Similarly, although the odds of owning health insurance cover decrease with household size, it is not significant. Nevertheless, other studies [20] find a significant relationship.

Being pregnant is associated with an odds ratio of a 1.19 increase in health insurance cover but is not significant at the 5% level. Yet reproductive health variable such as this one, has been established in other studies, at least in terms of the inverse relationship between maternity care and insurance, institutional delivery [21, 22]. In another study in Kenya [23], 86% of antenatal mothers interviewed indicated that they intended to pay for their delivery through insurance. Similarly [24] find that the implementation of a reproductive health voucher programme and a policy for free maternity services in Kenya was associated with an increase in facility delivery. Similar results obtain in Ghana [25] where women who had their ANC services covered had higher odds of attending at least one of the four ANC visits.

In the 2011 declaration of the UN General Assembly, Prevention and Control of Non-Communicable Diseases (NCDs) was brought to the global health agenda. In this declaration, essential medicines were identified as being central to treating chronic diseases such as hypertension and diabetes. A study to quantify access to essential medicines for people with chronic conditions in five low- and middle-income countries which included Kenya [26] finds that the likelihood of access to medicines for chronic diseases is elevated for those with medicines insurance coverage. It is lower among those who have a history of borrowing money to pay for medicines. The study documents limited access to essential medicines for chronic conditions in the five resource-constrained settings. It also shows the need for financial risk protection about generic medicines in the world’s efforts towards improving the treatment of chronic diseases. Other studies in Kenya [2] nevertheless find high and significant odds ratios of owning health insurance cover associated with having a chronic disease.

### Limitation

A number of social determinant variables such as educational level, marital status, and occupation could not be included as descriptive statistics and in the regression model due to data incompleteness. Similarly, variables related to health status such as known disability were equally not included in the model for the same reasons. In such cases, regression coefficients may be biased [27, 28]. For this study, it means that the true coefficients and odds ratios could be higher than or lower than outputs reported in Table 3 above. It will therefore be necessary to accurately and completely capture all the variables indicated in the household registration form (MOH 513).

## Conclusion

Health insurance coverage among women aged 15–49 years and who are either spouse of a household head or single mothers who are heads of households themselves is 11%, slightly higher than the 7% reported for the similar age group for the Western region of Kenya in the 2014 KDHS. Social determinants – age, perceived condition of the household, and wealth ranking are significantly related with use of a health insurance cover. Analysis of community household registration data presents a new opportunity to compare estimates for health which have traditionally been obtained from national sample surveys to the neglect of small and local areas such Navakholo sub-county. Further longitudinal research in the form of a repeat household registration should be conducted in the same households to determine the effect of in the NHIF recruitment drives. Similarly, stakeholders should organize for training of data collectors for community health, upstream and downstream, to ensure quality data are captured.

## Data Availability

Data extraction forms for this study are attached as Additional file 1 and Additional file 2. The data used in this study are not publicly available. This is because they were collected by a non-governmental organization – Amref Health Africa - which is a data controller as defined in the Kenya Government Data Protection Act No. 24 of 2019 (https://www.odpc.go.ke/dpa-act/). This Act requires that the data controller should get authorization from the Ministry of Health in order to provide access to the data to entities outside the country. Nevertheless, the anonymized data as well as STATA code are available with the corresponding author (rachel.ambalu@amref.org; racobiero@gmail.com) who will be able to mediate authorization for access to the data upon request.

## Abbreviations

ANC: Ante natal care
CHUs: Community Health Units
CHVs: Community Health Volunteers
iPUSH: innovative partnership for universal and sustainable healthcare
MJAli: mobile-Jamii Afya Link
MOH 513: Ministry of Health form 513
NCD: Non-communicable diseases
NGOs: Non-governmental organizations
NHIF: National Hospital Insurance Fund
NSNP: National Safety Net Programme
PBF: Performance-based financing
RMNCH: Reproductive, maternal, and newborn health
UHC: Universal Health Coverage
UM: Upper medium ecological zone
WHO: World Health Organization
WRAs: Women of reproductive age Abbreviations
